# Crosstalk between Human Microvascular Endothelial Cells and Tubular Epithelial Cells Modulates Pro-Inflammatory Responses Induced by Shiga Toxin Type 2 and Subtilase Cytotoxin

**DOI:** 10.3390/toxins11110648

**Published:** 2019-11-07

**Authors:** Romina S. Álvarez, Carolina Jancic, Nicolás Garimano, Flavia Sacerdoti, Adrienne W. Paton, James C. Paton, Cristina Ibarra, María M. Amaral

**Affiliations:** 1 Laboratorio de Fisiopatogenia, Departamento de Fisiología, Instituto de Fisiología y Biofísica Bernardo Houssay (IFIBIO Houssay-CONICET), Facultad de Medicina, Universidad de Buenos Aires, Buenos Aires 1121, Argentina; roalvarez_4@yahoo.com.ar (R.S.Á.); garimano@gmail.com (N.G.); flasacerdoti@gmail.com (F.S.); cristinaadrianaibarra@gmail.com (C.I.); 2 Laboratorio de Inmunidad Innata, Instituto de Medicina Experimental (IMEX-CONICET), Academia Nacional de Medicina, Buenos Aires 1425, Argentina; cjancic@hotmail.com; 3 Departamento de Microbiología, Parasitología e Inmunología, Facultad de Medicina, Universidad de Buenos Aires, Buenos Aires 1121, Argentina; 4 Research Centre for Infectious Diseases, Department of Molecular and Biomedical Science, University of Adelaide, Adelaide 5005, Australia; adrienne.paton@adelaide.edu.au (A.W.P.); james.paton@adelaide.edu.au (J.C.P.)

**Keywords:** Shiga toxin, Subtilase cytotoxin, Hemolytic Uremic Syndrome, endothelial/epithelial human renal cells, co-cultures, cytokines

## Abstract

Hemolytic uremic syndrome (HUS) is a consequence of Shiga toxin (Stx)-producing Escherichia coli (STEC) infection and is the most frequent cause of acute renal failure (ARF) in children. Subtilase cytotoxin (SubAB) has also been associated with HUS pathogenesis. We previously reported that Stx2 and SubAB cause different effects on co-cultures of human renal microvascular endothelial cells (HGEC) and human proximal tubular epithelial cells (HK-2) relative to HGEC and HK-2 monocultures. In this work we have analyzed the secretion of pro-inflammatory cytokines by co-cultures compared to monocultures exposed or not to Stx2, SubAB, and Stx2+SubAB. Under basal conditions, IL-6, IL-8 and TNF-α secretion was different between monocultures and co-cultures. After toxin treatments, high concentrations of Stx2 and SubAB decreased cytokine secretion by HGEC monocultures, but in contrast, low toxin concentrations increased their release. Toxins did not modulate the cytokine secretion by HK-2 monocultures, but increased their release in the HK-2 co-culture compartment. In addition, HK-2 monocultures were stimulated to release IL-8 after incubation with HGEC conditioned media. Finally, Stx2 and SubAB were detected in HGEC and HK-2 cells from the co-cultures. This work describes, for the first time, the inflammatory responses induced by Stx2 and SubAB, in a crosstalk model of renal endothelial and epithelial cells.

## 1. Introduction

Hemolytic uremic syndrome is clinically characterized by thrombocytopenia, non-immune hemolytic anemia, and acute renal failure (ARF) [1]. The most frequent presentation of HUS is the infection by Shiga toxin (Stx)-producing *Escherichia coli* (STEC) [2]. HUS is widely distributed throughout the world and studies about the global incidence of human STEC infections and deaths estimated that STEC causes more than 2.8 million acute illnesses annually, leading to 3,890 cases of HUS, 270 cases of end-stage renal disease and 230 deaths [3]. STEC O157:H7 has been the most frequent serotype associated with large outbreaks or sporadic cases of hemorrhagic colitis and HUS cases, although non-O157 serotypes have been increasingly reported to account for HUS [4]. Like in many parts of the world, in the southern countries of the continent (Argentina, Chile, Uruguay) STEC O157:H7 is associated with a significant number of HUS cases [5]. In Argentina, Stx-associated HUS is endemic and around the last 10 years, approximately 400 new cases were reported annually [6]. The incidence ranges since 10 to 17 cases per 100,000 children under 5 years of age, and the lethality is between 1 and 4% [7]. HUS is extremely prevalent in Argentina, being the most frequent cause of ARF and the second most important cause of chronic renal failure (CRF) in the pediatric age [8,9].

STEC O157:H7 and non-O157:H7 strains carry inducible lambda phages integrated into their genomes and can be transmitted between related bacteria [10]. The toxin genes are transcribed only during the lytic stage of the phage. The production of Stx is linked to the replication cycle of Stx phages, and the release of Stx is dependent on the lytic phase, which is induced under stress conditions [11]. The phages encode two types of Stx, Stx1 and Stx2. Stx2 presents several different variants (Stx2a to Stx2g), which have high homology (93–100%), except for Stx2f variant (69% identity) [12]. Furthermore, two additional Stx2 types, Stx2h and Stx2i, were recently described [13,14]. STEC strains encoding Stx2 are more frequently related to the most severe cases of HUS [15] and the subtype Stx2a cause more serious illnesses than strains encoding Stx2c [16].

Humans be able to be infected by STEC as a result of ingestion of raw meat, unpasteurized dairy products, contaminated water and vegetables, or direct contact with farm animals [17]. In addition, humans can become infected by transmission from person to person through the fecal-oral route [6]. This last infection pathway has become more prevalent in recent years and is favored by the low infective dose of STEC (< 100 bacteria). The possibilities of infection are associated with biological and cultural causes of the host, reservoirs and microorganism characteristics [18].

After infection, bacteria colonize the intestine and release Stx inside the lumen. Following this, Stx can access the systemic blood circulation and then contact the principal target cells by binding to the globotriaosylceramide (Gb3) receptor [19,20]. Stx is then internalized, by receptor mediated endocytosis and causes cell damage by the inhibition of protein synthesis and the induction of cell stress response pathways which finally unchains apoptosis [21].

Subtilase cytotoxin (SubAB) has also been associated with HUS pathogenesis as it was identified, in Australia, in a STEC strain belonging to the O113:H21 serotype, responsible for an outbreak [22] and it also was detected in isolates from bloody diarrhea HUS and diarrhea and household HUS contact in Argentina [23]. This cytotoxin damages cells through proteolytic cleavage of the endoplasmic reticulum chaperone BIP (GRP78) [24] and consequently, activates a massive ER (endoplasmic reticulum) stress response that finally ends in apoptosis [25,26,27]. To date, SubAB has not been detected yet in patients, but several serotypes of STEC expressing SubAB have been associated to HUS cases around the world [23]. Injection of purified SubAB has also been shown to stimulate the hallmark symptoms of HUS in mice [28].

SubAB binds α-2-3-linked *N*-glycolylneuraminic acid (Neu5Gc) through its pentameric B-subunit [22,29] and these Neu5Gc terminating glycans are not expressed on healthy tissues of humans [30]. However, these receptors can be incorporated by ingestion of red meat as well as dairy products [31].

So far, it is not well known how SubAB contributes to HUS pathophysiology; however, it was proposed that it is able to increase the clinical features of STEC infection [32].

It is well established that the kidney is seriously affected in Stx-associated HUS, because of the presence of especially Stx-sensitive cells that express high amounts of Gb3 receptor [33]. The thrombotic microangiopathy lesion is the typical injury caused by Stx in the kidney as a consequence of the direct action of Stx on glomerular endothelial cells and tubular epithelial cells by activating cellular death by apoptosis. In addition, Stx also can induce the release of pro-inflammatory cytokines, leukocyte recruitment, platelet aggregation and fibrin deposition. These events lead to partial or complete vessel occlusion by microthrombi and the consequent microangiopathic hemolytic anemia. Exeni et al. had postulated that inflammation could contribute to the endothelial damage [34]. Finally, kidney injury is expressed as different degrees of renal failure [35].

Most studies related to the Stx2 and SubAB effects in the kidney have employed renal endothelial and epithelial cell monocultures. However, the crosstalk established in vivo between these types of cells has not been considered. Accordingly, it has been postulated that the injury of renal tubules observed in patients with HUS is induced by the damage caused to the glomerular filtration barrier and enhanced by a direct action of Stx2 on the tubules [36,37]. In the kidney, proximal tubular cells and microvascular endothelial cells, both sensitive to Stx2 and SubAB [38,39], cooperate to regulate the hemodynamic and tubular function [40]. In this sense, we have previously developed a crosstalk model of human renal microvascular endothelial cells (HGEC) with human proximal tubular epithelial cells (HK-2) that attempted to simulate in vitro the physiological function of the human renal proximal tubule. In this crosstalk model, we found that Stx2 and SubAB cause different effects on cell viability and water absorption properties of HGEC/HK-2 co-cultures respect to HGEC and HK-2 monocultures. Contrary to the effects seen in monocultures, toxins did not cause inhibitory effects on water absorption across HGEC/HK-2 co-cultures and in addition, Stx2 cytotoxic effects on the monocultures’ viability were attenuated [39]. Considering these observations, we can speculate that soluble mediators secreted from endothelial and/or epithelial cells might be involved in these differential toxin effects. So, the purpose of this work was to analyze the response of pro-inflammatory cytokines released by HGEC and HK-2 monocultures compared to HGEC/HK-2 co-cultures exposed to Stx2 and SubAB, and try to shed light on the role of soluble mediators in regulating damage induced by Stx and/or SubAB in the kidney.

## 2. Results

### 2.1. Monocultures and Co-Cultures Exhibit Differential Secretion of Interleukin (IL)-6, Interleukin (IL-8) and Tumor Necrosis Factor (TNF)-α under Basal Conditions

**Figure 1:**
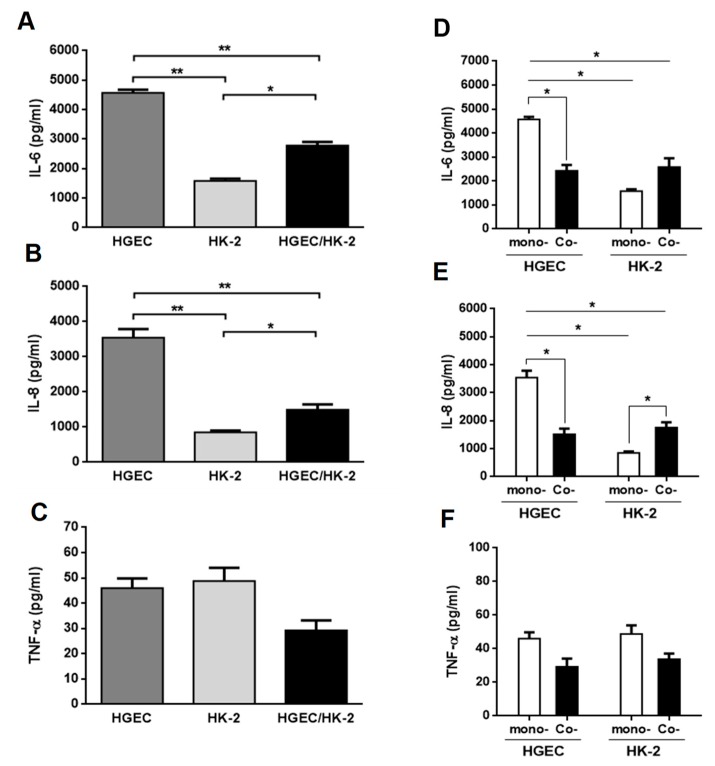
Cytokines secreted under basal conditions by HGEC and HK-2 monocultures and HGEC/HK-2 co-cultures. Basal levels of IL-6 (**A**), IL-8 (**B**) and TNF-α (**C**) secreted in supernatants of HGEC and HK-2 monocultures or in total supernatant of HGEC/HK-2 co-cultures were quantified by ELISA. Comparison are shown between IL-6 (**D**), IL-8 (**E**) and TNF-α (**F**) levels secreted by monocultures (mono-) of HGEC or HK-2 and HGEC or HK-2 co-culture compartments (Co-). Results are expressed as mean ± SEM of four experiments. HGEC vs. HK-2 vs. HGEC/HK-2 and HGEC or HK-2 co- vs. HGEC or HK-2 mono-, (*n* = 4). * *p* < 0.05, ** *p* < 0.01.

We evaluated the secretion of IL-6, IL-8 and TNF-α in HGEC and HK-2 monoculture supernatants and HGEC/HK-2 co-culture total supernatant, under basal conditions. We found that HGEC monocultures released a higher IL-6 and IL-8 concentration compared to HK-2 monocultures and HGEC/HK-2 co-cultures. In addition, the secretion of these cytokines by HGEC/HK-2 was also significantly higher compared to HK-2 (Figure 1A,B). On the contrary, HGEC/HK-2 co-cultures did not show any significant differences in TNF-α secretion compared to HGEC and HK-2 (Figure 1C). We then analyzed the secretion of IL-6, IL-8 and TNF-α by co-cultures, but quantifying cytokine production in each compartment, and compared these results to those obtained in HGEC and HK-2 monocultures. Figure 1D,E show that IL-6 and IL-8 secretion was higher in HGEC monocultures compared to the level observed on the HGEC side of co-cultures. Furthermore, while IL-8 release was higher on the HK-2 side of the co-culture relative to HK-2 monocultures (Figure 1E), non-significant differences were observed in IL-6 secretion (Figure 1D). Finally, TNF-α release did not show any significant differences between HGEC and HK-2 co-culture compartments compared to monocultures (Figure 1F).

### 2.2. Stx2 and SubAB Induce Changes in the Release of Pro-Inflammatory Mediators by HGEC and HK-2 Monocultures

**Figure 2:**
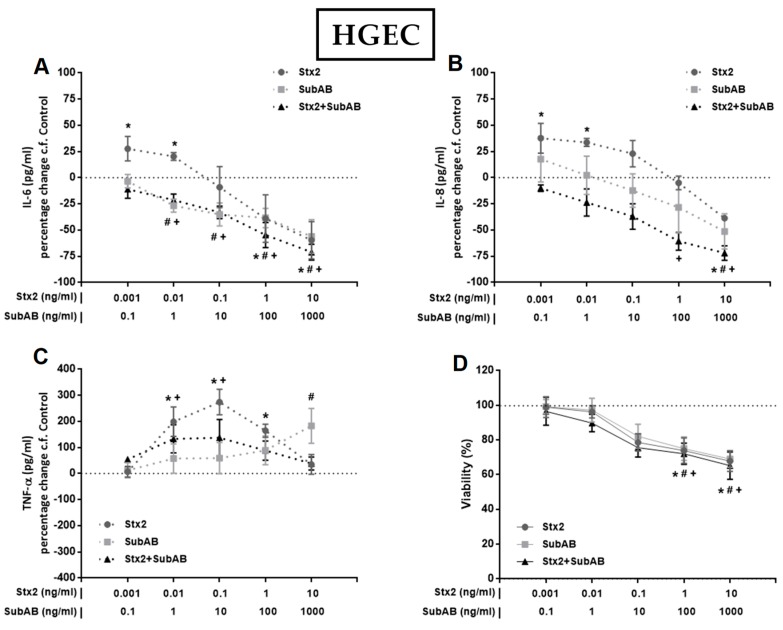
Effects of Stx2, SubAB and Stx2+SubAB on cytokine secretion and cell viability of HGEC monocultures. HGEC monocultures were treated with different concentrations of Stx2, SubAB and Stx2+SubAB for 24 h and IL-6 (**A**), IL-8 (**B**) and TNF-α (**C**) were quantified by ELISA. Results are expressed as the percentage (%) change in pg/mL relative to basal cytokine levels (control) (percentage change c.f Control). In addition, cell viability was analyzed by neutral red uptake (**D**). Absorbance was read at 540 nm and 100% was considered the viability of cells incubated under the same conditions but without toxin treatment (control). Results are expressed as mean ± SEM of three experiments. Ctrl vs. *Stx2, ^#^SubAB or ^+^Stx2+SubAB. *p* < 0.05.

**Figure 3:**
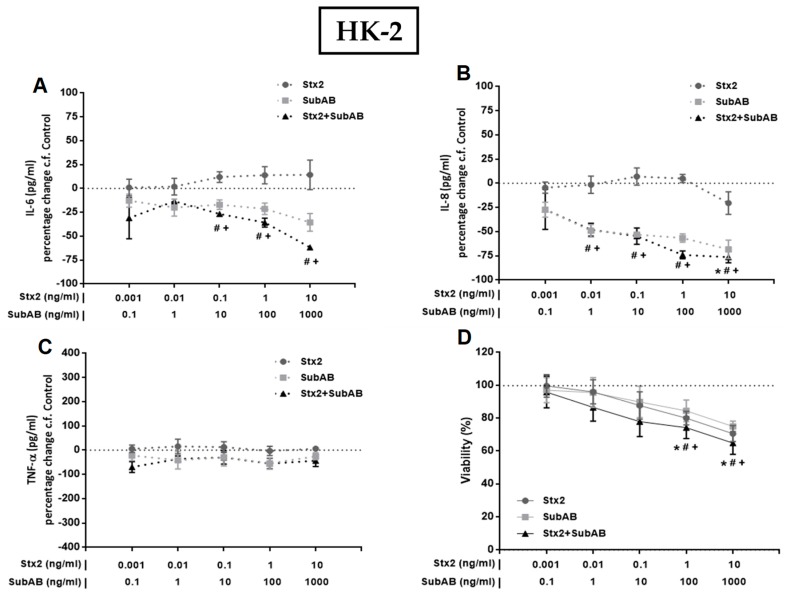
Effects of Stx2, SubAB and Stx2+SubAB on cytokine secretion and cell viability of HK-2 monocultures. HK-2 monocultures were treated to different concentrations of Stx2, SubAB and Stx2+SubAB for 24 h and IL-6 (**A**), IL-8 (**B**) and TNF-α (**C**) were quantified by ELISA. Results are expressed as the percentage (%) change in pg/mL relative to basal cytokine levels (control) (percentage change c.f Control). In addition, cell viability was analyzed by neutral red uptake (**D**). Absorbance was read at 540 nm and 100% was considered the viability of cells incubated under the same conditions but without toxin treatment (control). Results are expressed as mean ± SEM of three experiments. Ctrl vs. *Stx2, **^#^**SubAB or **^+^**Stx2+SubAB. *p* < 0.05.

To investigate the effect of Stx2 and SubAB on the production of pro-inflammatory cytokines by renal endothelial and epithelial cells, we first analyzed the secretion of IL-6, IL-8 and TNF-α by monocultures of HGEC and HK-2 after 24 h of treatment with different concentrations of Stx2, SubAB or Stx2+SubAB. We observed that high concentrations of Stx2 (10 ng/mL), SubAB (100 and 1000 ng/mL) and co-incubation with both toxins induced a significant decrease in the secretion of IL-6 and IL-8 by HGEC (Figure 2A,B). Likewise, when we analyzed the secretion of these cytokines by stimulated HK-2 cells, we observed a similar effect to that found in HGEC (Figure 3A,B). In addition, Stx2 (1 and 10 ng/mL), SubAB (100 and 1000 ng/mL) and co-incubation with both toxins, were able to reduce the cell viability of HGEC (Figure 2D) and HK-2 (Figure 3D). In contrast to previous results, the lowest Stx2 concentrations used (0.001–1 ng/mL) increased the secretion of IL-6, IL-8 and TNF-α by HGEC; and SubAB at 1000 ng/mL or co-incubation with Stx2 + SubAB (0.01 ng/mL + 1 ng/mL; 0.1 ng/mL + 10 ng/mL) increased the secretion of TNF-α (Figure 2A–C). HK-2 cells only showed inhibitory effects of the toxins on the IL-6 and IL-8 secretion or no effect on the release of TNF-α (Figure 3A–C).

### 2.3. Stx2 and SubAB Exhibit Differential Effects on IL-6, IL-8 and TNF-α Secretion by Monocultures and Co-Cultures

**Figure 4:**
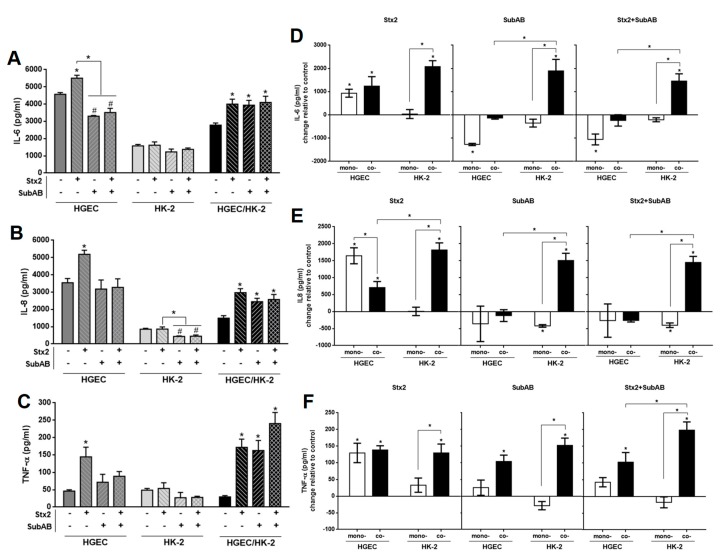
Differential effects on IL-6, IL-8 and TNF-α secretion by monocultures and co-cultures exposed to Stx2, SubAB and Stx2+SubAB. HGEC, HK-2 monocultures and HGEC/HK-2 co-cultures were exposed to Stx2 (0.01 ng/mL), SubAB (1 ng/mL) or Stx2+SubAB (0.01 ng/mL + 1 ng/mL) for 24 h. IL-6 (**A**), IL-8 (**B**) and TNF-α (**C**) secreted in supernatants of HGEC and HK-2 monocultures or in total supernatant of HGEC/HK-2 co-cultures were quantified by ELISA. Comparison between IL-6 (**D**), IL-8 (**E**) and TNF-α (**F**) levels secreted by monocultures (mono-) of HGEC or HK-2 and HGEC or HK-2 co-culture compartments (Co-). Results are expressed as mean ± SEM of four experiments. HGEC vs. HK-2 vs. HGEC/HK-2 and HGEC or HK-2 co- vs. HGEC or HK-2 mono-, (*n* = 4). * *p* < 0.05.

Next, we evaluated the secretion of IL-6, IL-8 and TNF-α in HGEC and HK-2 monocultures and HGEC/HK-2 co-cultures after exposure to Stx2 (0.01 ng/mL), SubAB (1 ng/mL) or Stx2+SubAB, added into the HGEC compartment. We selected the toxin concentrations that did not affect HGEC and HK-2 cell viability and that were able to stimulate cytokine secretion by HGEC. After 24 h of incubation, all treatments increased the secretion of IL-6, IL-8 and TNF-α by co-cultures relative to controls (Figure 4A–C). In addition, when we compared the release of IL-6 in monocultures with co-culture compartments, we found that Stx2 increased IL-6 in the HGEC side similarly to that observed in HGEC monocultures (Figure 4D). On the other hand, SubAB and Stx2+SubAB did not induce any significant change in the secretion of IL-6 in the HGEC compartment with respect to untreated cells. Unlike in HGEC monocultures, a significant decrease in the concentration of IL- 6 was observed (Figure 4A,D). When the HK-2 compartment was compared to HK-2 monocultures, we observed that Stx2, SubAB and Stx2+SubAB did not modify the concentration of IL-6 in HK-2 monocultures (Figure 4A,D); in contrast, the toxins induced a significant increase of this cytokine production in HK-2 compartment of co-cultures (Figure 4D). Finally, in co-cultures, the IL-6 concentration exhibited significant differences for SubAB and Stx2+SubAB treatments between the endothelial and epithelial compartments (Figure 4D).

On the other hand, Stx2 caused a significant increase in IL-8 production in the HGEC compartment when cultured together with HK-2 and in HGEC monocultures relative to controls (Figure 4B,E). Interestingly, the IL-8 concentration was higher in HGEC monocultures than in the HGEC compartment when co-cultured with HK-2 cells (Figure 4E). In addition, non-significant differences were observed in SubAB and Stx2+SubAB treatments (Figure 4B,E). In relation to epithelial cells, Stx2, SubAB and Stx2+SubAB significantly increased IL-8 in the HK-2 compartment of co-cultures compared to controls and also to HK-2 monocultures (Figure 4E). Moreover, a decrease in IL-8 secretion was observed in HK-2 monocultures by SubAB and Stx2+SubAB and non-significant changes were detected for Stx2 treatment (Figure 4B,E).

Moreover, TNF-α secretion was significantly increased by Stx2, SubAB and Stx2+SubAB treatments in the endothelial and epithelial co-culture compartments compared to controls (Figure 4F). We found that Stx2 increased TNF-α in the HGEC compartment similarly to that observed in HGEC monocultures (Figure 4F). Furthermore, a significant increase in TNF-α secretion by Stx2, SubAB and Stx2+SubAB was observed in the HK-2 compartment with respect to HK-2 monocultures, where no modulation had been detected after treatment with the toxins (Figure 4C,F). Similar results were obtained when comparing the release of TNF-α in the HGEC compartment relative to HGEC monocultures after SubAB and Stx2+SubAB treatment (Figure 4F).

### 2.4. Soluble Mediators Released by HGEC Increase the Secretion of IL-8 by HK-2

**Figure 5:**
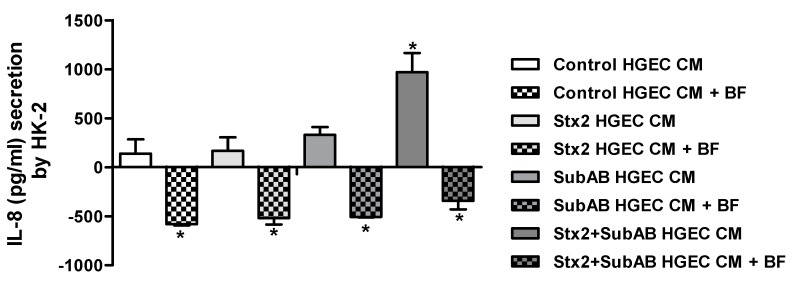
IL-8 secretion by HK-2 cells exposed to HGEC conditioned media. HK-2 cells were incubated during 24 h with CM of HGEC previously treated or not with Stx2 (0.01 ng/mL), SubAB (1 ng/mL) or Stx2+SubAB (0.01 ng/mL + 1 ng/mL) for 24 h and in presence or not of BF. IL-8 concentration was quantified by ELISA. IL-8 concentration = (IL-8 concentration in the supernatant of HK-2 after each treatment) – (basal IL-8 concentration in the supernatant of control HK-2) – (IL-8 concentration in the CM of control or treated HGEC). Results are expressed as mean ± SEM of three experiments. Ctrl vs. Stx2, SubAB or Stx2+SubAB and Ctrl or treatments vs. Ctrl or treatments + BF, *p < 0.05.

Taking into account the results obtained regarding the differential secretion of cytokines induced by toxins in the HK-2 co-culture compartment compared to HK-2 monocultures, we hypothesize that soluble factors secreted by HGEC in the co-culture could stimulate the secretion of IL-6, IL-8 and TNF-α by HK-2. As shown in Figure 5, the conditioned medium (CM) derived from HGEC control, Stx2, SubAB and Stx2+SubAB treatments were able to augment the secretion of IL-8 by HK-2 monocultures. In addition, a significant increase was observed when cells were stimulated with CM from HGEC treated with Stx2+SubAB relative to CM of control HGEC. Furthermore, when Brefeldin A (BF) was incorporated to HK-2 monocultures after the addition of HGEC CM, a blockage of IL-8 secretion by HK-2 monocultures was obtained for all treatments (Figure 5). On the contrary, we did not observe any modulation for IL-6 and TNF-α secretion (data not shown).

### 2.5. Toxins Were Incorporated by HGEC and HK-2 Cells Derived from Co-Cultures

**Figure 6:**
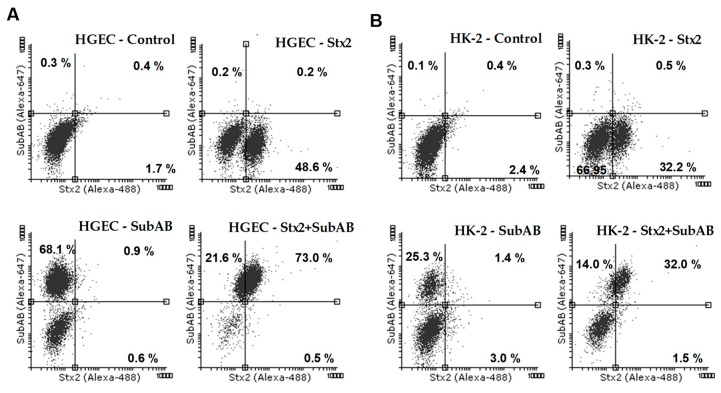
Internalization of Stx2, SubAB or both toxins by HGEC and HK-2 in co-culture. HGEC/HK-2 co-cultures were exposed or not to Stx2 (0.01 ng/mL), SubAB (1 ng/mL) or Stx2+SubAB (0.01 ng/mL +1 ng/mL) for 24 h. Then HGEC and HK-2 cells were removed from the cell culture inserts and the presence of Stx2, SubAB and Stx2+SubAB were evaluated by HGEC and HK-2 intracellular staining and flow cytometry analysis. Stx2 was detected by a mouse anti-Stx2 and then with an anti-mouse Dylight 488-conjugated antibody. SubAB was identified by a rabbit anti-SubAB and then with an anti-rabbit Alexa Fluor 647-conjugated antibody. The results are expressed as the percentage of positive cells for Stx2, SubAB and Stx2+SubAB. Representative dot plots are shown for HGEC (**A**) and HK-2 (**B**).

Finally, we analyzed the presence of Stx2 and SubAB in HGEC and HK-2 cells recovered from the two different sides of the co-culture. In the HGEC co-culture compartment, Stx2 was detected in about 48% of HGEC cells, SubAB in 63% and both toxins in 68% (Figure 6A, Table 1). In addition, in the HK-2 co-culture compartment, Stx2 was detected in around 35% of HK-2 cells, SubAB in 32% and both toxins in 41% (Figure 6B, Table 2).

## 3. Discussion

STEC strains are recognized by their ability to cause severe disease in humans, like HUS. Pianciola & Rivas [4] have reported the incidence of STEC O157 infections and HUS cases in different countries. These data show that Argentina exhibits the highest prevalence of HUS in the world, probably related to high circulation of hypervirulent clade 8 strains [41,42].

This pathology cannot be easily prevented and to date, there is no authorized vaccine or effective therapy is available for humans. Therefore, extension of our knowledge about Stx2 and SubAB action and their deleterious effects in the kidneys, is critical to find a strategy to control this disease. Previously, we have developed an in vitro human proximal tubule model. In this model we had shown that Stx2 and SubAB effects were attenuated when endothelial and epithelial renal cells were cultured in a very close proximity, unlike in isolated endothelial and epithelial monocultures [39]. Therefore, we proposed that these differential effects could be due to a special microenvironment containing soluble factors produced during the communication between microvascular endothelial cells and proximal tubular cells. Furthermore, in recent years, several studies in both in vitro and in vivo models have demonstrated the activation of an inflammatory response that favors the cytokines secretion, the kidney leukocytes recruitment and the activation of coagulation and complement cascades during the development of HUS. In this sense, patients with Stx-associated HUS have shown immunological parameters that are indicative of an inflammatory state [34]. Taking into account the importance of the inflammatory microenvironment in HUS, in this work we analyzed the secretion of selected pro-inflammatory mediators released by HGEC/HK-2 co-cultures and HGEC and HK-2 monocultures, at basal conditions and after treatment with Stx2, SubAB or with both toxins together (Stx2 + SubAB).

Our results showed that cytokine secretion, in basal conditions, depends on the type of cells and whether those cells were cultured as monocultures or cocultures. The basal concentration of IL-6 has been reported in two different models of co-culture and in correlation with our results, a difference in IL-6 concentration was observed between monocultures and co-cultures. In these studies, no additive effect of microvascular endothelial cells and tubular epithelial cells was found for the secretion of IL-6 [43,44].

We studied cytokine secretion in monocultures and showed that high concentrations of Stx2, SubAB or Stx2+SubAB decreased IL-6 and IL-8 secretion by HGEC monocultures and reduced the cell viability, as we had previously reported [38,39]. Nevertheless, low concentrations of Stx2 that have no effect on cell viability, induced an increase in the secretion of these cytokines. In addition, both toxins individually or co-incubated, also increased the secretion of TNF-α.

Our results obtained in HGEC monocultures are in accordance with those previously reported in other models. In this regard, we can mention that Stx induced the up regulation of IL-8, IL-6, MCP-1 and GM-CSF in several endothelial cell lines, at concentrations that are non-inhibitory for protein synthesis and non-cytotoxic in terms of cell viability [45,46,47,48,49,50]. MCP-1 and IL-8 were up regulated by Stx2 in glomerular endothelial cells [46]. In addition, Stx2 induced the released of IL-8, Regulated upon Activation, Normal T cell Expressed, and Secreted (RANTES), Stromal Cell-Derived Factor 1 alpha (SDF-1α), and Stromal Cell-Derived Factor 1 beta (SDF-1β) by the microvascular endothelial cell line HMEC-1 [45].

In relation to SubAB, and similar to our results, Wang et al. showed that SubAB decreased the secretion of IL-8 and MCP-1 in a colonic epithelial cell line (HCT-8) and in brain microvascular endothelial cells (HBMEC); in this latter cell line they also observed a reduction in IL-6 release. Additionally, they found a discrepancy between mRNA and protein levels suggesting that the destruction of BiP by SubAB action caused a disruption of ER function that compromises cytokine export [50].

Regarding the co-incubation of both toxins together (Stx2 + SubAB), there is very little information available. It has been reported by Wang et al. that the treatment of the human macrophage U937 cell line with both toxins together avoided the stimulatory effect of Stx2 on the secretion of IL-8 [50]. This observation is in agreement with our results obtained in HGEC monocultures, in which we observed that while Stx2 stimulated the IL-8 release, co-incubation with both toxins had no effect or even decreased their production. Therefore, in accordance with these results, SubAB could modulate the inflammatory response induced by Stx2. Contrary to HGEC monocultures, we found that Stx2 did not modulate the release of any of these pro-inflammatory cytokines by HK-2 monocultures. SubAB and the co-incubation with Stx2 decreased IL-6 and IL-8 secretion, but had no effect on the release of TNF-α. In this regard, Lentz et al. demonstrated that HK-2 cells treated with Stx1 and Stx2 increased the TNF-α and IL-1 mRNA levels without a simultaneous increase in expression of the respective protein [51].

In this work, we used SubAB concentrations two orders of magnitude higher than those of Stx2. We have previously demonstrated that different concentrations of Stx2 and SubAB are required to obtain similar HGEC and HK-2 cell damage effects [38,39]. Taking into account these observations, we can speculate that the higher concentrations of SubAB, when compared to Stx2, required to elicit cell damage is probably due to the low abundance of Neu5Gc-terminating receptor structures in HGEC and HK-2, although we can’t exclude their incorporation from the FCS in the medium.

On the other hand, co-cultures incubated with Stx2, SubAB, or both toxins, showed an increase in cytokines secretion. So, co-cultures treated with these toxins showed the opposite effect with respect to HGEC monocultures where we found that SubAB and both toxins did not stimulate or suppress IL-6, IL-8 and TNF-α release.

A lower concentration of IL-6 and IL-8 was observed in the endothelial compartment of the co-culture compared to HGEC monocultures, under basal conditions. Previous reports suggested that differences may be related to the activation of primary cultures of microvascular endothelial cells when they are cultured as monolayers [52] and a special microenvironment generated in the co-cultures could be responsible for decreasing the basal endothelial activation [43,53].

Surprisingly, after incorporation of Stx2, SubAB and both toxins on the co-culture endothelial side, a significant increase of IL-6, IL-8 and TNF-α was observed on the epithelial (HK-2) side compared to HK-2 monocultures. Taking this into account, we could hypothesize that in this co-culture model, when toxins are incorporated on the endothelial side, they stimulate the release of IL-6, IL-8 and TNF-α in both endothelial and epithelial compartments. In addition, a greater increase of these cytokines was evident in the epithelial compartment of the co-culture relative to the endothelial compartment, as well as in comparison with the HGEC and HK-2 monocultures. In this sense, Bijuklic et al. demonstrated in a co-culture model that after treatment with LPS, an increase of IL-8 and IL-6 in the epithelial side was observed, without changes in the endothelial side [44].

To clarify this point, we studied if soluble mediators released by HGEC monocultures that had been incubated or not with toxins, could stimulate HK-2 monocultures for the secretion of cytokines. We found that HK-2 monocultures released a higher concentration of IL-8 after treatment with CM of HGEC and this effect was more pronounced after treatment with CM of HGEC previously incubated with both toxins. In addition, we corroborated that this cytokine had been produced by HK-2 cells according to Brefeldin A treatment. Taking into account that HK-2 cells express TNF-α receptors [54], in our co-culture model, we can speculate that the epithelial cells could produce inflammatory mediators as a consequence of sensing TNF-α released by HGEC cells.

Interestingly, Stx2 and SubAB were detected in HGEC and HK-2 cells after co-culture. Importantly, the presence of each toxin in the HK-2 cells was observed even though they were not incubated in direct contact with Stx2 and SubAB and, no decrease in the cell viability was observed. It is possible that the access of Stx2 and SubAB from the HGEC compartment to the HK-2 compartment is favored by changes in the endothelial paracellular pathway as a result of the secretion of some cytokines. In this regard, previous reports on human lung microvascular endothelial cells show that TNF-α promotes the opening of endothelial intercellular gaps. [55,56]. Furthermore, after treatment with Stx2+SubAB, most of cells were positive for both toxins. Taking into account that Stx2 and SubAB have different receptors, both located in the cell membrane within specific domains [57], it is possible to speculate that the interaction of one toxin with its receptor facilitates the binding of the other toxin to its own, e.g., by upregulation of the cognate glycans. To clarify this point, further studies will be necessary.

In summary, we demonstrate that endothelial-epithelial communication contributes to the inflammatory response stimulated by the action of Stx2 and SubAB toxins on renal cells. In this sense, we propose that after STEC infection, toxins can access the blood stream and bind the endothelial cells and also the epithelial cells. When high toxin concentrations are circulating, they cause direct cell damage in renal epithelial and endothelial cells that should be attenuated due to the close-proximity between these cells, as we previously showed in vitro [39]. However, when low toxin concentrations are circulating, they do not cause direct cell damage or a decrease in the cell viability, but Stx2 may induce the release of soluble pro-inflammatory mediators such as IL-6, IL-8 and TNF-α by endothelial cells. In addition, the very close proximity between the endothelial and epithelial cells could allow TNF-α released from endothelial cells to stimulate epithelial cells to release IL-6, IL-8 and TNF-α, thereby amplifying the inflammatory process in the proximal tubule.

## 4. Conclusions

Data from this work describe, for the first time, the inflammatory response stimulated by Stx2 and SubAB in a co-culture model of human renal endothelial and epithelial cells. This inflammatory response could contribute to the initial events of ARF observed in patients with HUS.

## 5. Materials and Methods

### 5.1. Reagents

Purified Stx2a was provided by Phoenix Laboratory, Tufts Medical Center, Boston, MA, USA. Lipopolysaccharide (LPS) content on Stx2 was checked by Limulus amebocyte lysate (LAL) test (<10 pg/mL). SubAB was purified from recombinant *E. coli* by Ni-NTA chromatography via a His_6_ tag fused to the C-terminus of the B subunit, as described previously [22]. Purity was greater than 98%, as judged by SDS-PAGE and staining with Coomassie Blue.

### 5.2. Antibodies

Mouse monoclonal antibody 2E11 against the A-subunit of Stx2 was kindly provided by Dr. Roxane M. F. Piazza [58]. Rabbit anti-SubA has been described previously [59]. Anti-mouse Dylight 488-conjugated and anti-rabbit Alexa Fluor 647-conjugated antibodies were purchased from Invitrogen (ThermoFisher Scientific, Buenos Aires, Argentina); rabbit anti-VWF was from DAKO (Tecnolab, Buenos Aires, Argentina); mouse anti-CD31 (PECAM-1) −FITC was from Sigma Aldrich.

### 5.3. Human Primary Glomerular Microvascular Endothelial Cell Culture

Human glomerular endothelial cells (HGEC) were isolated from kidney fragments removed from normal areas from different pediatric patients with segmental uropathies or tumors in one pole and normal creatinine that were undergoing nephrectomies performed at Hospital Nacional “Alejandro Posadas”, Buenos Aires, Argentina (written informed consent was obtained from the next of kin, caretakers, or guardians on the behalf of the minors/children participants involved in our study, nº: 035 LUP1So/17 (13)). The Ethics Committee of the University of Buenos Aires approved the use of human renal tissues for research purposes. Endothelial cells were isolated as was previously described [38]. Once isolated, cells were grown in M199 media supplemented with 20% fetal calf serum (FCS), 3.2 mM L-glutamine, 100 U/mL penicillin/streptomycin (GIBCO, Waltham, MA, USA) and 25 μg/mL endothelial cell growth supplement (ECGS, Sigma, St. Louis, MO, USA) as previously reported [38]. For growth-arrested conditions, a medium with a half of FCS concentration (10%) and without ECGS was used. For the experiments, cells were used between 2–7 passages, after characterization for von Willebrand factor and platelet/endothelial cell adhesion molecule 1 (PECAM-1) positive expression [38].

### 5.4. Tubular Human Epithelial Cell Line Culture

Human proximal tubular epithelial cell line (HK-2) was purchased from American Type Culture Collection (ATCC, Manassas, VA, USA) and grown in DMEM/F12 medium (Sigma Aldrich, St. Louis, MO, USA) containing 10% FCS, 100 U/mL penicillin/streptomycin (GIBCO, Waltham, MA, USA), 2 mM L-glutamine (GIBCO, Waltham, MA, USA), 15 mM HEPES at 37 °C in a humidified 5% CO_2_ incubator. For growth-arrested conditions, medium without FCS was used.

### 5.5. Monocultures and Co-Cultures of Renal Endothelial and Epithelial Cells

Co-cultures of HGEC and HK-2 cells were performed using Millicell cell culture inserts (PIHP01250, Millipore, Billerica, MA, USA). HGEC cells (5.10 ^4^) were seeded on the lower side of the filter (0.4 μm membrane pore size) and allowed to attach for 12–16 h. Then, inserts were inverted and HK-2 (7.10 ^4^) cells were seeded into the upper side. Co-cultures were maintained in HGEC complete medium. For the epithelial and endothelial monocultures, the same procedure was carried out with the exception that partner cells were not added [39]. To evaluate the effects of Stx2 and SubAB on monocultures and co-cultures, Stx2 or SubAB were added to the lower compartment (HGEC compartment).

### 5.6. Integrity of Endothelial and Epithelial Monolayers and Bilayers

The integrity of endothelial and epithelial monolayers and bilayers was checked by using a Millicell-ERS electric resistance system (Millipore, Billerica, MA, USA) calibrated for each measurement, as previously described [39]. For that, the electrical resistance (TEER) was monitored daily during the development of cell culture until confluence was achieved. At this time, TEER values were stabilized, indicating that HGEC and HK-2 monolayers and HGEC/HK-2 bilayers had reached confluence.

### 5.7. Cytokine Detection by ELISA

Basal levels of IL-6, IL-8 and TNF-α cytokine secretion were measured in HGEC, HK-2 monoculture and co-culture supernatants. Cytokines released in co-cultures were quantified in the total supernatant (HGEC+HK-2 supernatants) and in the HGEC and HK-2 co-culture compartment supernatants, separately. Monocultures of HGEC and HK-2 were incubated or not during 24 h with different concentrations of Stx2 (0.001–10 ng/mL) or SubAB (0.1–1000 ng/mL) or both Stx2 + SubAB. For controls, growth-arrested conditioned medium was used.

For comparisons between monocultures and co-culture regarding pro-inflammatory cytokine secretion after toxin treatment, Stx2 (0.01 ng/mL), SubAB (1 ng/mL) or Stx2 (0.01 ng/mL) + SubAB (1 ng/mL) were employed.

Cell culture supernatants were collected and IL-6, IL-8 and TNF-α were quantified by ELISA, according to the manufacturer’s recommendations. Human IL-6, IL-8 and TNF-α ELISA kits were from BD Bioscience (San Diego, CA, USA).

For analyzing the effects of conditioned media [54] of HGEC on HK-2 cell secretion of IL-6, IL-8 and TNF-α, HGEC monocultures were treated or not with Stx2 (0.01 ng/mL), SubAB (1 ng/mL) or Stx2 (0.01 ng/mL) + SubAB (1 ng/mL) during 24 h, then CM was collected and employed to incubate HK-2 cells with them for 24 h. In addition, Brefeldin A was incorporated or not for blocking the HK-2 cells ER to Golgi transport, 1 h after HGEC CM incubation to estimate the contribution of HK-2 on cytokine release. The concentration of cytokines secreted by HK-2 monocultures after exposure to HGEC CM was calculated as follows: (cytokine concentration in the HK-2 supernatant after each treatment)—(basal cytokine concentration in the control HK-2 supernatant)—(cytokine concentration in the CM of control or treated HGEC). Results are expressed as cytokine concentration in pg/mL.

### 5.8. Cell Viability Evaluation

To evaluate the Stx2 and SubAB effect on the cell viability, the neutral red cytotoxicity assay was adapted from previously described protocols [60]. Briefly, HGEC and HK-2 monocultures were treated or not during 24 h with Stx2 (0.001–10 ng/mL) or SubAB (0.1–1000 ng/mL) or both Stx2 + SubAB, in growth arrested conditions. After treatment, freshly diluted neutral red (10 µg/mL, Sigma Aldrich, St. Louis, MO, USA) was added to a final concentration of 10 μg/mL and cells were incubated for an additional 1 h at 37 °C in 5% CO_2_. Cells were then washed and fixed with 200 μL 1% CaCl_2_ + 1% formaldehyde and then lysed with 200 μL 1% acetic acid in 50% ethanol to solubilize the neutral red. Absorbance in each well was measured in an automated plate spectrophotometer at 540 nm. Results were expressed as percentage of cell viability, where 100% represents cells incubated under identical conditions but without toxins.

### 5.9. Intracellular Detection of Stx2 and SubAB by Flow Cytometry in HGEC/HK-2 Co-Cultures

The presence of Stx2 and SubAB incorporated by the cells was evaluated in HGEC and HK-2 co-culture compartments by flow cytometry. For that, after treatment, cells were fixed in 1% paraformaldehyde and permeabilized with 0.1% saponin in phosphate-buffered saline (PBS). Then, cells were incubated in PBS 0.5% FCS with mouse anti-Stx2 for 2 h and then with a secondary anti-mouse Dylight 488-conjugated antibody for 1 h, or incubated with rabbit anti-SubAB for 2 h and then with a secondary anti-rabbit Alexa Fluor 647-conjugated antibody for 1 h. Cells were subsequently washed twice and resuspended in PBS. The staining was analyzed by flow cytometry on PARTEC PAS III using Flowing software 2.5.1.

### 5.10. Data Analysis

Data are presented as mean ± SEM. Statistical analysis was performed using Graph Pad Prism Software 5.0 (San Diego, CA, USA). ANOVA was used to calculate differences between groups and Tukey’s multiple comparisons test was used a *posteriori*. Statistical significance was set at *p* < 0.05.

## Figures and Tables

**Table 1:** HGEC co-culture compartment. Positive cells for Stx2, SubAB and Stx2+SubAB, means ± SEM, *n* = 3. Ctrl vs. Stx2, SubAB or Stx2+SubAB, * *p* < 0.05.

HGEC	Control		Stx2		SubAB		Stx2+SubAB	
	%	SEM	%	SEM	%	SEM	%	SEM
**Stx2 +**	1.5	1.2	48.4 *	8.5	0.7	0.4	0.9	0.4
**SubAB +**	0.2	0.1	0.9	1.3	62.6 *	7.5	20.3 *	4.1
**Stx2 – SubAB +**	0.3	0.1	0.3	0.2	0.7	0.6	68.3 *	4.2

**Table 2:** HK-2 co-culture compartment. Positive cells for Stx2, SubAB and Stx2 + SubAB, means ± SEM, *n* = 3. Ctrl vs. Stx2, SubAB or Stx2+SubAB, * *p* < 0.05.

HK-2	Control		Stx2		SubAB		Stx2+SubAB	
	%	SEM	%	SEM	%	SEM	%	SEM
**Stx2 +**	1.8	1.1	35.0 *	4.1	1.9	0.9	4.9	6.6
**SubAB +**	0.3	0.1	3.8	4.4	31.8 *	5.9	12.5 *	2.0
**Stx2 – SubAB +**	0.4	0.1	0.8	0.4	0.9	0.4	40.6 *	15.3
